# Engineering megakaryocytes with oncolytic viruses for *in situ* generation of therapeutic platelets

**DOI:** 10.1016/j.omton.2026.201184

**Published:** 2026-04-09

**Authors:** Yinxian Yang, Zhen Gu, Jicheng Yu

**Affiliations:** 1 State Key Laboratory of Advanced Drug Delivery and Release Systems, School of Pharmacy, Zhejiang University, Hangzhou 310058, China; 2 Fujian Provincial Key Laboratory of Innovative Drug Target Research, State Key Laboratory of Vaccines for Infectious Diseases, Xiang An Biomedicine Laboratory, School of Pharmaceutical Sciences, Xiamen University, Xiamen 361102, China; 3 Jinhua Institute of Zhejiang University, Jinhua 321299, China

Oncolytic viruses that selectively lyse tumor cells and elicit the innate antitumor immune response represent a highly attractive approach for cancer therapy.^1^ However, the clinical application of oncolytic viruses primarily involves intratumoral injection, which is not only complex to perform but also difficult to reach inaccessible tumors and metastatic sites.^2^ Intravenous administration enables systemic delivery of oncolytic viruses, extending their therapeutic potential from primary tumors to widespread metastatic lesions while improving patient compliance and clinical convenience. However, neutralizing antibodies and immune clearance lead to rapid elimination of viral particles following intravenous injection, accompanied by off-target distribution and systemic inflammatory responses.^1^^,^^3^ Therefore, the development of strategies to achieve efficient and safe tumor-targeted delivery of oncolytic viruses via intravenous administration is crucial for their clinical application and optimal therapeutic outcomes.

Platelets, serving as key sentinels in the immune defense system, are frequently hijacked by viral pathogens, transforming them from immune effector cells into viral hitchhikers and shelters.^4^ For example, despite antiviral treatment, replication-competent human immunodeficiency virus (HIV) can still be recovered from platelets, indicating the existence of a platelet-mediated “Trojan horse” transport mechanism that enables the virus to evade immune clearance.^4^ Notably, platelets play a crucial role in cancer metastasis by recognizing circulating tumor cells and adhering to them to form cell aggregates.^5^ Furthermore, platelets actively contribute to hemostasis and angiogenesis, enabling them to efficiently target postoperative tumor beds while also specifically locating tumor-associated neovascularization and metastatic foci.^5^^,^^6^ Although platelets have been explored for anticancer drug delivery, the active internalization of large particles for cytoplasmic loading poses challenges in anucleate platelets compared to nucleated mammalian cells. Furthermore, receptor-mediated endocytosis of viral particles may trigger platelet activation, which can convert resting platelets into a pre-activated state.^7^ Platelets are anucleate cell fragments shed from mature megakaryocytes during differentiation. Consequently, exogenous genes or viral particles incorporated into megakaryocytes can be transferred and retained in the subsequent platelet population during thrombopoiesis process.^6^ Recent studies also show that in patients with COVID-19, SARS-CoV-2-infected pulmonary megakaryocytes promote systemic viral dissemination and pulmonary inflammation by releasing virus-containing platelets into the circulation.^8^

Inspired by the natural mechanisms of thrombopoiesis and the systemic spread of SARS-CoV-2 via platelet carriage, we recently reported in *Proceedings of the National Academy of Sciences of the United States of America* an approach in which megakaryocytes were engineered with oncolytic viruses to generate oncolytic platelets *in vivo*.^9^ This strategy can not only evade rapid clearance of the oncolytic virus by pre-existing neutralizing antibodies and other antiviral immune responses but also enhance tumor-targeting efficiency by leveraging the physiologic interaction of platelets with cancer cells. To harness megakaryocyte-derived platelets as Trojan horse carriers, we induced mature megakaryocytes from both the murine megakaryocytic progenitor L8057 cell line and primary bone marrow cells, characterized by polyploid nuclei, high expression of CD41/CD42, and the ability to generate platelets. Oncolytic adenovirus type 5 was then efficiently loaded into mature megakaryocytes with a loading efficiency as high as 98.5%, without impairing the inherent platelet-producing capacity of megakaryocytes. Of note, engineered megakaryocytes achieved high viral payload packaging, compared to the suboptimal viral particle load in donor platelets. We observed that approximately 90.2% of platelets derived from engineered megakaryocytes carried viral particles, and these oncolytic platelets exhibited no significant differences from native platelets in morphology, adhesion capacity, or functional phenotype.

Upon intravenous injection, engineered megakaryocytes released virus-carrying oncolytic platelets *in situ* under the shear stress microenvironment of pulmonary vessels. Upon contact with tumor cells, oncolytic platelets were activated, releasing oncolytic viruses and platelet-derived particles containing these viruses, which selectively infected tumor cells. We demonstrated that intravenous administration of engineered megakaryocytes effectively suppressed tumor growth in the A549 orthotopic lung cancer model, leading to significantly prolonged survival in mice. Furthermore, systemic administration of engineered megakaryocytes enhanced antitumor immune responses by remodeling the immunosuppressive tumor microenvironment and inducing T cell infiltration, thereby sensitizing tumors to PD-1/PD-L1 blockade therapy. Engineered megakaryocytes combined with PD-L1 antibodies markedly inhibited the growth of lung metastases in CT26 colon cancer, while significantly preventing postoperative recurrence and progression of lung metastases in B16F10 melanoma. We also found that intravenous injection of engineered megakaryocytes into mice with postoperative and metastatic tumors elicited tumor-specific T cells and memory T cells, thereby suppressing tumor growth and providing long-term immune protection.

In this work, we engineered megakaryocytes to efficiently encapsulate oncolytic viruses, thereby generating oncolytic platelets *in vivo* for systemic viral therapy. Such cascade cell carriage strategy significantly improved therapeutic outcomes of oncolytic viruses, meanwhile reducing off-targeted side effects of systemic viral delivery. Combination strategies are expected to further enhance the synergistic therapeutic potential of this platform. Beyond co-administration with existing immunotherapies such as PD-L1 antibodies, synthetic biology techniques can be integrated to enable megakaryocyte-based cell therapies. For example, by genetically engineering megakaryocytes to express therapeutic antibodies or immunomodulatory cytokines, the derived platelets can not only serve as drug carriers but also exert therapeutic effects as immunomodulators within the microenvironment. Despite the therapeutic potential demonstrated by megakaryocyte infusion, future studies should carefully evaluate the long-term safety and hematologic effects of megakaryocyte infusion, particularly regarding the potential risk of inducing procoagulant states and thrombosis in the hypercoagulable conditions commonly observed in cancer patients.

In summary, the megakaryocyte-platelet cascade delivery shielded oncolytic viruses from systemic clearance while enabling tumor-targeted delivery and responsive release. This platform is not only suitable for oncolytic virus delivery but can also be extended to deliver various therapeutic payloads, including small-molecule compounds, nucleic acids, proteins, and nanoparticles that are particularly difficult to load directly onto platelets. Compared to directly modifying donor platelets, engineered megakaryocytes not only achieve high viral loading efficiency but also enable *in vivo* mass production of therapeutic platelets, thereby reducing pre-activation of platelets during *ex vivo* processing. To advance the clinical translation of this platform, several key challenges remain to be addressed. For instance, although *ex vivo* expansion systems for megakaryocytes and platelets have been established by directed differentiation of pluripotent stem cells or hematopoietic stem cells,^10^ the differentiation efficiency, maturation status, and heterogeneity of megakaryocytes still require further improvement.

## Declaration of interests

Z.G. is the co-founder of Zcapsule Inc. and μZen Inc.
